# Supplementary material for: Gradient-Pattern Micro-Grooved Wicks Fabricated by the Ultraviolet Nanosecond Laser Method and Their Enhanced Capillary Performance
Source: Micromachines (Basel). 2024 Jan 22;15(1):165. doi: 10.3390/mi15010165 (PMC10819530; doi:10.3390/mi15010165)
Supplement: Supplementary file 1 [file micromachines-15-00165-s001.zip › micromachines-2821894-supplementary.pdf]

Supplementary Material

The capillary rising height is determined by the average height of the meniscus, which is indicated by the boundary of purple and orange color. Taking the third wick in Figure 12a as an example, the liquid level is uneven. The height of the white line is the average height of the meniscus. It meets the relationship of:

$$A_a = A_b + A_c$$

where A_a is the area of the purple region on the upper side of the white line, A_b and A_c are the areas of the orange regions below the white line. The advantage of this method is better accuracy and reliability by avoiding errors introduced by individual grooves.

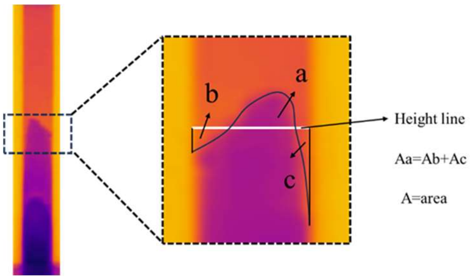

Figure S1. The operating criteria for determining the meniscus height.
